# Symposium on Pellagra

**Published:** 1917-10

**Authors:** J. C. Johnson

**Affiliations:** Atlanta


### Symposium on Pellagra.

DR. J. C. JOHNSON, Atlanta.

In this symposium, attention was directed to the following problems:

1. Theory of specific infection.
2. Theory of special means of conveyance, as by contagion, insects, etc.
3. Theories as to dietetic causes of special nature, non-drying oils, fermented corn, badly balanced diet, etc.
4. Theory that pellagra is not a specific infection nor due to special cause but is a state of poor nutrition, auto-intoxication, etc. Under this theory, can a special system-complex, marked by diagnostic signs and symptoms develop?

If our definition of pathology is correct, the symptom-complex of pellagra strongly suggests that specific infection plays an important role in its production. But if it is infectious the period of its incubation is anomalous, and there must preexist a condition of the body not congenitally a part, nor normally a product of vital action, and not usually present in infectious diseases. This latter, if true, explains why pellagra is not contagious, and also why efforts to transmit it have been unsuccessful.

The fact that we have not been able to isolate from the excreta of pellagrins bacteria of a pathogenic nature to which we might ascribe the disease cannot reasonably be accepted as proof that it is not infectious. There are many bacteria with which perhaps we are not familiar, or some of whose existence we may not know. The cause is not always manifest in a result. There are many bacteria in the intestines which in certain media are no doubt harmless, but which in other media may assume a virulent disposition. In 1911 we published the view that "Pellagra is a disease of perverted metabolism, having its first expression in the epithelial structures of the alimentary tract." Further observation has not changed our convictions in this particular. However, metabolism and digestion cannot be perfect or imperfect one without the other. Each is a sequence and a cause of the other and nutrition is the ultimate **condition** which results from their coordinate processes. Metabolism is but another name for the chemical and physical forces of the cells under the guidance of a directing agency. These forces have no self-control and cannot of their own accord change their relations, and hence cannot be the only agent in any disease. For these and other reasons we do not believe that any article of food or deficiency in any principle of food can cause pellagra. In a few isolated cases diet may appear to be a determining factor in its production, and a special diet may be beneficial in the treatment. But many who are using the most appropriate food are developing, and some of them are dying of pellagra. In any effort to explain the phenomena of pellagra by errors in diet, it should be remembered that a lack of equilibrium is not classical disease, and that in health, the laws that control the commerce of the body are so fixed that when one organic principle is lacking in the food, another can be converted to fill its place. The readiness with which the body can adjust itself to unusual demands, so often seen in instances of prolonged fasting, forced or voluntary, as well as in diseases, furnishes to us ample proof that pellagra means something more than deficiency of either of the proximate principles of the body.

---

MARVIN H. SMITH, Jacksonville, Fla.

I have studied quite a large number of cases of pellagra in both Georgia and Florida, and at the present time I feel fairly well convinced that the disease is brought about by wrong eating—not necessarily an unbalanced diet though the histories of most of my cases show that they have indulged in an abundant amount of cheap carbohydrate diet, a fair amount of fat bacon or other animal fat (not butter) and a very limited amount of animal proteins, legumes, fresh fruits and vegetables.

My belief is that pellagra is a disease arising not only from deficient proteins, but a deficiency of vitamins. To support my belief, my experience has convinced me that these cases make progress when proteins are added to their daily menu, but more satisfactory progress, when some fruit juices, whole grain products and puree of fresh mashed tender vegetables can be given.

I am of the opinion that ultimately we will find that pellagra belongs in the same category with scurvy and beriberi, in which the absence of certain vitamins are a more potent factor than the absence of any particular food element.

---

DR. J. T. ROGERS, Savannah, Ga.

First I do not believe that pellagra is caused by the taking of any special kind of diet or by the lack of any special kind of diet. Pellagra is undoubtedly influenced by diet just as tuberculosis is influenced by diet. (2) I believe this disease is caused by a specific organism or bacillus—possibly plasmodium.

Why do I believe this:—Because I have had patients from the same neighborhood. One "well to do" (wealthy) and **well nourished**, had plenty of the best food, **and ate it**, and another **poor** and **poorly nourished**, and while on the same medical treatment I've seen the well nourished "well to do" patient die in few months, while the poor and poorly nourished one got relief from all pellagra symptoms, and so far as we know may be cured.

We could write pages broadening and detailing, but you asked for answer in few words, and we feel that it is not necessary to go into detail.

---

**Maternal Mortality** From All Conditions Connected with Childbirth. Grace L. Meigs, Misc. Series No. 6, Children's Bureau, U. S. Dept. of Labor. From 100 to 1913, registration-area statistics show 5.3-7.1 deaths from puerperal septicaemia, per 100,000 population and 7.1-8.5 due to other conditions depending on pregnancy and childbirth. The statistics of the whole country for 1913, show that of deaths of females between the ages of 15 and 44, tuberculosis leads with 26,265; and that puerperal septicaemia and accidents connected with pregnancy and labor follow with 9,876 deaths. The next highest is heart disease, 6,386. Apparently, exact statistics do not exist for reducing these figures to terms of mortality per 100 or 1000 deliveries but they represent approximately 5 maternal deaths per 1000 deliveries. We have at other times, estimated that with due allowance for indirect causes of death depending on child birth, the average modern American woman would be killed by bearing 10 children. For other countries, for various recent periods of which statistics are available, the maternal deaths per 1000 births are as follows: Australia, puerperal septicaemia 1.8, other causes directly due to pregnancy and confinement 3.4; Austria, 1.9 and not stated; Belgium, 2.3 and 3.5; England and Wales, 1.6 and 2.4; France, 2.4 and 2.8; Hungary, 1 and 2.6; Ireland, 1.9 and 3.6; Italy, 1 and 1.7; Japan, 1.4 and 2.6; New Zealand, 1.1 and 3.3; Norway, 1.5 and 1.4; Prussia, 1.5 and 1.8; Scotland, 1.8 and 3.6; Spain, 3.6 and 2.1; Sweden, 1 and 1.4; Switzerland, 2.4 and 3.2.

---

**Gas in the Tissues.** J. D. Morgan, and G. Vilvandre, Radiographers for the Canadian and British General Hospitals, respectively, Brit, Med. Jour., Jan. 6, emphasize the value of X-rays in the diagnosis, especially in cases not presenting the usual symptoms and signs.

